# Automated and data-driven plate computation for presurgical cleft lip and palate treatment

**DOI:** 10.1007/s11548-023-02858-6

**Published:** 2023-04-02

**Authors:** Till N. Schnabel, Baran Gözcü, Paulo Gotardo, Lasse Lingens, Daniel Dorda, Frawa Vetterli, Ashraf Emhemmed, Prasad Nalabothu, Yoriko Lill, Benito K. Benitez, Andreas A. Mueller, Markus Gross, Barbara Solenthaler

**Affiliations:** 1 Department of Computer Science, ETH Zurich, 8092 Zurich, Switzerland; 2 DisneyResearch|Studios, 8006 Zurich, Switzerland; 3 Oral and Craniomaxillofacial Surgery, University Hospital Basel and University of Basel, 4031 Basel, Switzerland; 4 Department of Clinical Research, University of Basel, 4031 Basel, Switzerland; 5 Department of Biomedical Engineering, University of Basel, 4123 Allschwil, Switzerland

**Keywords:** Presurgical orthopedic plates, Cleft lip and palate, Automated digital design, Orthopedic treatment, Mesh landmark detection, Geometry processing

## Abstract

**Purpose:**

Presurgical orthopedic plates are widely used for the treatment of cleft lip and palate, which is the most common craniofacial birth defect. For the traditional plate fabrication, an impression is taken under airway-endangering conditions, which recent digital alternatives overcome via intraoral scanners. However, these alternatives demand proficiency in 3D modeling software in addition to the generally required clinical knowledge of plate design.

**Methods:**

We address these limitations with a data-driven and fully automated digital pipeline, endowed with a graphical user interface. The pipeline adopts a deep learning model to landmark raw intraoral scans of arbitrary mesh topology and orientation, which guides the nonrigid surface registration subsequently employed to segment the scans. The plates that are individually fit to these segmented scans are 3D-printable and offer optional customization.

**Results:**

With the distance to the alveolar ridges closely centered around the targeted 0.1 mm, our pipeline computes tightly fitting plates in less than 3 min. The plates were approved in 12 out of 12 cases by two cleft care professionals in a printed-model-based evaluation. Moreover, since the pipeline was implemented in clinical routine in two hospitals, 19 patients have been undergoing treatment utilizing our automated designs.

**Conclusion:**

The results demonstrate that our automated pipeline meets the high precision requirements of the medical setting employed in cleft lip and palate care while substantially reducing the design time and required clinical expertise, which could facilitate access to this presurgical treatment, especially in low-income countries.

**Supplementary Information:**

The online version contains supplementary material available at 10.1007/s11548-023-02858-6.

## Introduction

Cleft lip and palate is the most common craniofacial birth defect with a prevalence of approximately 1 in 700 live births worldwide [1]. Presurgical orthopedic (PSO) treatment is commonly used to narrow the cleft and align the alveolar segments over the course of a few months by inserting an individually designed plate into the neonate’s mouth, which keeps the tongue out of the cleft. As such, PSO treatment facilitates the surgeries and can decrease their number [2]. Additionally, the treatment reportedly facilitates feeding, improves the facial appearance by reducing the nasal deformity, and helps develop early normal phonology [3].

Since its introduction in the 1950s, PSO treatment has evolved into various types of therapies involving orthopedic devices ranging from active to passive plates. Whereas active plates, such as nasoalveolar molding devices [4], apply external forces to jointly mold the nose and lip while directing the alveolar segments into the ideal position, passive plates simply bridge the cleft space and prevent tongue pressure on the cleft edges, which causes the cleft to gradually narrow on its own (passively), solely due to the change in the intraoral balance of forces [3]. Conventionally, these plates are fabricated using plaster casts which are obtained from physical imprints taken within the first weeks after birth [2]. This impression-taking is resource intensive requiring highly specialized personnel, still bearing risks for respiratory obstruction [6]. Recent digital alternatives adopt intraoral scanners to replace this risky impression-taking and employ 3D printing to allow for a fully digital workflow [7–10]. However, some of these digital workflows point out the complexity of the employed 3D software, which therefore requires professional operators, and emphasized the importance of developing automated methods [9]. Grill et al. [11] first address this automation by offering a graphical user interface (GUI) called RapidNAM that is clinically analyzed for unilateral (UCLP) and later extended by Schiebl et al. [12] to bilateral (BCLP) cleft lip and palate cases. RapidNAM aligns the input scans by minimizing least squares objectives and segments the alveolar ridges based on maximum heights and empirically defined area thresholds. Given this segmentation, RapidNAM fits ellipses and polynomials to model the alveolar arches and premaxilla in order to compute a series of NAM plates in 10–15 min targeting a healthy anatomy. Since the initial alignment and segmentation do not always succeed and some plates contain artifacts from the input scan, Schiebl et al. conclude that their software should be used semi-automatically rather than automatically [12].

We propose an alternative method for segmentation and alveolar cleft bridging based on deep learning and nonrigid surface registration to remove such potential artifacts and to avoid the inherent limitations of segmentation based on alveolar ridge heights and empirically defined thresholding. Additionally, we focus on the passive plate type employed in [13] rather than NAM devices. Although the recommended inputs to our pipeline are 3D intraoral scans, scanned plaster casts are equally supported. The raw input scan is automatically landmarked via DiffusionNet [14] in a two-stage process to increase the prediction accuracy on arbitrarily oriented meshes. The landmarks are employed in the initial alignment of the given mesh scan with its corresponding template as well as in the subsequent registration, which yields a segmentation of the input mesh via the regions pre-segmented on the template. Based on this segmentation, a well-fitting plate is computed automatically via several mesh processing steps mainly comprising cleft filling and volumization using a custom curvature-selective Laplacian smoothing algorithm. We demonstrate the accurate landmark prediction on UCLP and BCLP cases as well as the proper fit and clinical feasibility of the automatically computed plates, which are fabricated with a 3D printer at the point-of-care. The resulting workflow is time- and resource-efficient, offers increased infant safety, and is accessible to doctors via a custom GUI. Our main contributions can be summarized as follows:

- A novel and fully automated pipeline for presurgical orthopedic plate computation with generic applicability to both unilateral and bilateral types of cleft lip and palate.
- Substantial speed-up and facilitation of the digital design process compared to the state-of-the-art.
- A 3D deep-learning-based landmark prediction applied to palatal scans of arbitrary mesh topology and orientation using two sequential neural networks for an increased precision.
- Validation of our pipeline’s medical applicability via a study based on printed models, where 12 out of 12 plates were approved by two cleft care specialists, and via a preliminary clinical evaluation, where 19 patients have started treatment with our automated designs.

**Fig. 1:**
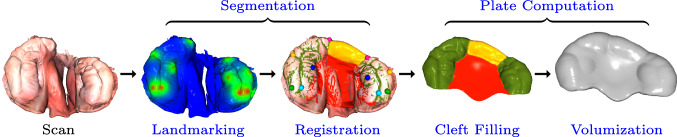
Visualization of our pipeline given a unilateral cleft lip and palate intraoral scan as input. The landmarks are automatically extracted from neural segmentation map predictions. They initially guide the nonrigid surface registration of a template that bridges over the scan’s alveolar cleft, as marked in yellow between the two anterior pink landmarks. This registration yields a segmentation of the input scan, thus enabling the automated plate computation, which comprises filling of the cleft palate area (central region colored in red) and volumizing the surface to be 3D-printable

## Materials and methods

We present an AI-driven method and a fully automated workflow for the computation of presurgical orthopedic plates given a unilateral (UCLP) or bilateral (BCLP) cleft lip and palate scan as input. An overview of the pipeline is illustrated in Fig. 1. We also refer the reader to our supplementary material for additional implementation details (Online Resource 1) and for a visual summary of our software (Online Resource 2).

### Segmentation

We employ a deep-learning-based landmark prediction and a subsequent surface registration to segment a palate scan of arbitrary mesh topology and orientation, thus eliminating any laborsome manual preprocessing steps.

#### Landmarking

**Fig. 2:**
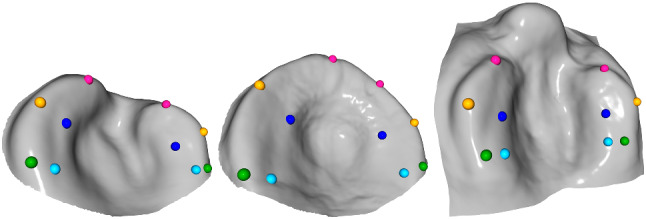
Template designs for the cleft types UCLP (left), isolated palate (middle), and BCLP (right). Each template was modeled as an average of multiple palatal meshes of the corresponding cleft type and landmarked in an equal fashion as the input scans

We collected a dataset comprising 283 UCLP and 114 BCLP scans of patients mostly aged between 0 and 8 months. Old plaster cast imprints were digitized via 3D scanning, whereas new patient data were captured with an intraoral scanner.^1^ For each cleft type, we modeled a template as an average of multiple palatal meshes of the corresponding cleft type and annotated them with a set of 10 landmarks ${\mathcal {L}}_t$, as illustrated in Fig. 2. To initially guide the template registration via sparse correspondences (cf. “Registration” section), we manually applied the same landmark annotation scheme ${\mathcal {L}}_g$ to each scan in our dataset. As such manual landmarking is slow and requires the involvement of an experienced user, we then trained the recent DiffusionNet model from Sharp et al. [14] on a subset of our data to predict these landmark positions on arbitrary cleft lip and palate meshes. Hence, new input scans can be processed efficiently and fully automatically by our pipeline. Since Sharp et al. do not discuss the problem of landmarking, we adopt the DiffusionNet extension from [15] and represent each landmark as a sparse vertex segmentation map, but we change the fall-off around each landmark to the following continuous exponential:

1 $$\begin{aligned} s_{i,j} = \exp (-0.5 \Vert {\textbf{l}}_j - {\textbf{v}}_i\Vert _2), \end{aligned}$$

where $s_{i,j} \in [0, 1]$ denotes the segmentation value for vertex ${\textbf{v}}_i$ on the input scan given landmark ${\textbf{l}}_j \in {\mathcal {L}}_g$. The 3D positions are defined in (mm). Such smooth functions essentially model artificial probability maps for the landmark positions, which facilitates training compared to one-hot user annotations that are inherently imprecise; cf. Fig. 1 for a visualization of the segmentation maps.

Unlike [15], we trained a DiffusionNet model with randomized rotations to support input meshes of arbitrary orientation. The model uses the 3D vertex coordinates ${\textbf{v}}_i$ as input features without additional color information. The actual landmark positions ${\mathcal {L}}_{p_1}$ are extracted from the model prediction via an argmax operation over the vertex dimension. Additionally, we compute the model’s prediction certainty ${\mathcal {P}}_1$ by normalizing its output to [0, 1] range. For an arbitrarily oriented input mesh, this first model prediction forms the course initialization for our second prediction step. The input mesh is aligned with the template of its corresponding cleft type via weighted Procrustes analysis [16] applied between the template landmarks ${\mathcal {L}}_t$ and the predicted landmarks ${\mathcal {L}}_{p_1}$ weighted by their prediction probability ${\mathcal {P}}_1$. A second model is then applied to the aligned mesh for a more accurate landmark prediction. This second model was trained on the same dataset with the difference that the meshes were all pre-aligned using our manual landmark annotations ${\mathcal {L}}_g$ and varied only by a limited azimuthal range of ± 0.2 rad around the *z*-axis during training. The fact that the model was trained on this limited rotation range makes it more precise in its prediction ${\mathcal {L}}_{p_2}$ and corresponding probability ${\mathcal {P}}_2$, but still robust enough towards small alignment errors caused by the less precise landmark prediction ${\mathcal {L}}_{p_1}$ of the first model. For the UCLP and BCLP cleft types, separate models were trained with the mean squared error (MSE) loss on 80% of their corresponding dataset until they converged on the remaining test data after approximately 500 epochs, which took less than a day on an NVIDIA RTX 2080 TI. All models utilize the default parameters proposed for segmentation purposes by Sharp et al. [14], except for the dropout percentage set to 0.2. We provide more training details in the supplementary (Online Resource 1), and present the results in the “Experiments and results” section.

#### Registration

Having aligned the input scans with their corresponding template, we apply the nonrigid iterative closest point (NICP) registration algorithm introduced by Amberg et al. [17] in order to determine a dense correspondence between the template and each input scan. This algorithm iteratively deforms the template to minimize its distance to the input scan while considering multiple constraints, including an optional landmark term to initially guide the registration with the sparse correspondences defined between the landmarks ${\mathcal {L}}_t$ and ${\mathcal {L}}_{p_2}$. We extend this constraint with a per-landmark weighting given by the prediction probability ${\mathcal {P}}_2$ from the DiffusionNet model. Additionally, a stiffness constraint ensures a moderately uniform triangle distribution where local holes are filled, and since the template has a fixed vertex topology, the input scans are all effectively resampled to the template’s resolution. We adopt the NICP implementation from Booth et al. [18] with the main adjustment that only the alveolar ridges (colored in green in Fig. 1) are registered instead of the whole template in order to shift the registration focus towards the area relevant for the contact region of the plate. The remaining regions are registered in a subsequent cycle with the alveolar ridges serving as a fixed constraint. Furthermore, to bridge over the alveolar cleft for UCLP cases, we modeled an additional template averaged over a set of isolated cleft palate scans that exhibit a healthy alveolar ridge (cf. Fig. 2), and we annotated the UCLP alveolar cleft region on it to be excluded from deforming towards the input scan while still serving as a stiff constraint during optimization. After registration, this region consequently simulates a corresponding healthy alveolar ridge structure instead of following the course of the alveolar cleft. For NAM plates, the same procedure could be adopted to cover the premaxilla in BCLP scans by bridging over both alveolar clefts. Figure 1 visualizes this alveolar bridging for our passive UCLP plates in the region colored in yellow between the two most anterior landmarks. Finally, the dense correspondence computation automatically yields a segmentation of the input scan, since the various regions pre-segmented on the template represent the corresponding structures of the scan after its registration. This segmentation, also visualized in Fig. 1, enables the automated mesh processing required for the plate computation described next.

### Plate computation

A plate is computed automatically with the registered scan data via a cleft filling and a volumization step.

#### Cleft filling

The cleft is eventually closed with surgery. To narrow the cleft presurgically, the ideal plate is designed such that it covers the alveolar ridges closely. In the cleft palate region, however, the design should rather keep a considerable gap to the cleft tissue, allowing for inwards growth, while simulating the curved shape of a healthy palate to offer the tongue as much room as possible [19]. Hence, after cutting out a pre-defined plate area from the registered scan, we imitate such a healthy palate shape by smoothing the vertices in the cleft palate region around a sphere that connects the left and right alveolar ridge and approximates the course of the cleft palate region. This cleft approximation is constrained to maintain a user-adjustable safety distance to the cleft tissue with the empirically determined default value of 2.5 mm. The sphere parameters are computed via an optimization of a constrained least squares objective, which is described in more detail in the supplementary (Online Resource 1).

#### Volumization

**Fig. 3:**
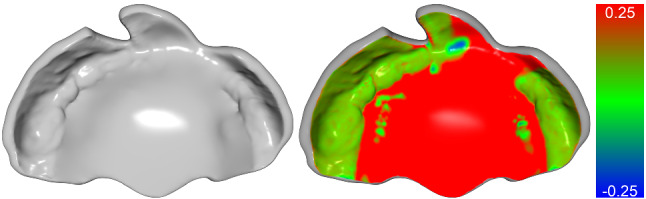
High-frequency geometric details are preserved in the plate’s contact area, while smooth surfaces are achieved in the non-contact cleft palate region. The colors indicate the signed point-to-plane distance between the plate and the input scan in (mm)

The surface with the filled cleft palate region needs to be volumized to meet the required final plate thickness of approximately 2 mm to avoid instability or breaking in thin areas. Hence, we developed a curvature-selective smoothing algorithm, where convex regions are selectively smoothed out before offsetting the vertices along their normals to avoid creating large self-intersections. This “convex smoothing” algorithm, described in more detail in the supplementary (Online Resource 1), is applied to two copies of the plate surface. The first copy represents the plate’s contact side and is offset by 0.1 mm to compensate for potentially slight inaccuracies in the plate’s contact region around the alveolar ridges. The second copy is offset by an additional 2 mm and connected with the first copy via a half-ellipse between the corresponding boundary points to obtain a smooth border transition. Together, the connected surfaces form a volumized mesh with a thickness optionally adjustable in different areas; e.g., the thickness around the buccal borders (close to the patient’s cheeks) may be reduced to avoid plate destabilization. Other user options include the addition of a ventilation hole and stimulation elements for possibly improved speech development [8]. Finally, selective smoothing adjustments of empirically determined intensity ensure that the plate does not cause any abrasion in the mouth of the infant while avoiding large discrepancies between the original palatal structure and the plate’s contact region. Figure 3 visualizes the contact side of a sample plate to demonstrate the selectively preserved high frequency details in the contact region.

## Experiments and results

Our pipeline is implemented in Python and uses Blender for the GUI, where optionally several parameters can be customized, such as the plate thickness or distance to the palatal cleft (see Online Resource 1). The GUI was tested and default parameters were empirically determined in accordance with the requirements specified by multiple cleft care professionals. We validate our pipeline on our test set with quantitative measurements and qualitative assessments. After digital comparisons, medical feedback is incorporated to assess a set of 3D-printed models. Lastly, we describe the application of our software in a preliminary clinical evaluation.

### Quantitative evaluation

**Table 1:** Landmark prediction distance and signed point-to-plane distance between our computed plates and their corresponding input mesh in the contact region

		Distance (mm)
Landmark prediction	UCLP	$1.69 \pm 1.85$
	BCLP	$1.70 \pm 1.28$
Plates w/ manual landmarks ${\mathcal {L}}_g$	UCLP	$0.1 \pm 0.07$
	BCLP	$0.1 \pm 0.12$
Plates w/ learned landmarks ${\mathcal {L}}_{p_2}$	UCLP	$0.09 \pm 0.15$
	BCLP	$0.09 \pm 0.09$

The results are solely based on the test data. Plates with manual landmarks require medical expertise and manual labor, whereas our learned landmarks result in a fully automated computation

We first evaluate the accuracy of the learning-based landmark prediction ${\mathcal {L}}_{p_2}$ and then analyze the sensitivity of the landmarks on the resulting geometrical shape of the plate. The measurements are summarized in Table 1. Our learning-based approach achieves an average landmark prediction distance of less than 2 mm for both cleft types. We compute the signed point-to-plane distance of the final plates to their corresponding input scans, with a negative sign referring to a point on the plate lying inside the input, which may imply a pressure point. The contact distance distribution, also visualized in Fig. 3, is closely centered around the targeted 0.1 mm lift-off, regardless whether the landmarks are positioned manually (Plates w/ manual landmarks ${\mathcal {L}}_g$) or automatically (Plates w/ learned landmarks ${\mathcal {L}}_{p_2}$), suggesting that the computed plate shapes fit tightly. However, although these quantitative distance measurements provide a practical way to assess the landmark prediction accuracy and plate tightness, the significance of these results might be limited for clinical usability. Therefore, we provide further evaluation in the following subsections.

### Evaluation of printed models

**Fig. 4:**
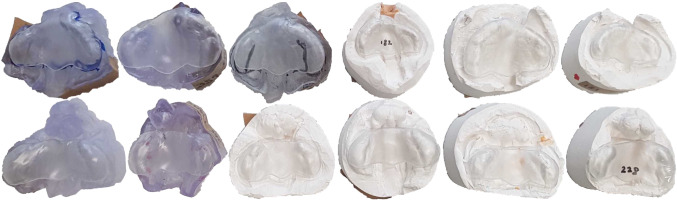
Automatically computed and 3D-printed plates given twelve different intraoral scans and plaster cast impressions—top UCLP, bottom BCLP. Two cleft care professionals assessed each plate’s suitability based on its shape and its fit on the corresponding palate model

We selected 6 UCLP and 6 BCLP meshes from our test set—4 plaster cast impressions and 2 intraoral scans for BCLP and 3 of both for UCLP. The selection per cleft type was randomized and confirmed by cleft experts to be a suitable representation of the variety of possible models. We automatically computed plates for each of the selected models. In a first step, we digitally compared the Plates w/ manual landmarks ${\mathcal {L}}_g$ to the Plates w/ learned landmarks ${\mathcal {L}}_{p_2}$ qualitatively by rating a random permutation of the two variants to avoid any decision bias. No significant preference could be determined towards the Plates w/ manual landmarks ${\mathcal {L}}_g$, verifying that our two-stage DiffusionNet setting is sufficiently accurate for the remaining pipeline. Afterwards, the plates and their corresponding palate scans were 3D-printed using a medically-approved biocompatible material^2^ (USP Class VI certified). An orthodontist assessed 10 out of 12 (5 UCLP and 5 BCLP) printed plates as suitable for use on patients without any adjustment, whereas the remaining 2 were estimated to be usable after minor subtractive corrections. Furthermore, a surgeon assessed all 6 UCLP and 1 BCLP plate as perfectly fitting and ready for use on patients, whereas the remaining 5 BCLP plates were presumed to be applicable after small subtractive adjustments in the buccal area and close to the premaxilla. All 12 printed and assessed plates are presented in Fig. 4. These experimental results imply that our pipeline is able to compute reliable plates in a fully automated fashion.

**Fig. 5:**
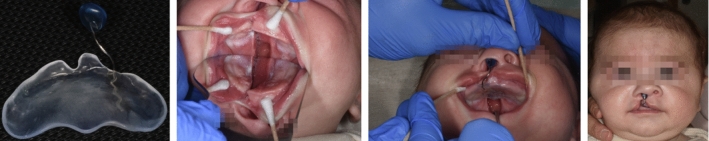
A UCLP plate is presented along with its use on a patient. From left to right, we show the printed plate with the nasal stent added post-print, two close-up shots of the patient’s palatal cleft (one accentuated by a mirror in the mouth, one with the inserted plate), and finally a picture of the patient with the inserted plate

### Preliminary clinical evaluation

Following these results, our pipeline has been implemented in clinical routine at the University Hospital Basel, Switzerland and Saveetha Medical College & Hospital, Chennai, India, where digital intraoral scanning has already reduced the conventional impression-taking time from over 60 min to less than 20 min [10]. Our automated method has further reduced the time required for the subsequent plate fabrication, estimated at 45 min for hands-on manufacturing on the plaster cast model and at 35 min for manual CAD modeling time [10], to a maximum of 5 min, consisting only of 1–2 min of manual involvement and an automated runtime of approximately 3 min. Moreover, since the manual involvement only requires simple mesh import/export operations, the plate modeling job, which previously required a plate specialist to operate complex CAD software, could be assigned to an assistant, thus saving significant costs during plate fabrication. As a result, 19 patients (14 UCLP and 5 BCLP) have started or already finished treatment in the specified hospitals with our automated plates. 9 out of the 19 plates were grinded for minor adjustments, which is also common for conventionally fabricated plates, and nasal stents were manually added post-print. None of the plates caused any harmful pressure points or abrasion. Figure 5 shows photographs of the first patient being treated with our automated plate at the University Hospital Basel. We are planning to conduct a broader clinical study to evaluate the impact of this new plate computation software on the overall treatment outcome.

## Conclusion and future work

We presented a data-driven pipeline for fully automated presurgical orthopedic plate computation. Our pipeline considerably speeds up the design time compared to the state-of-the-art without requiring any user expertise for the approximately 3 min of automated computations. Moreover, our DiffusionNet [14] adaptation successfully predicts the required landmarks on meshes of arbitrary orientation with sufficient precision, and we showed that the subsequent classical registration allows for domain-specific adjustments, such as gap bridging. Lastly, two medical experts assessed 12 out of 12 3D-printed plates computed by our pipeline as usable either immediately or after minor subtractive grinding, and a total of 19 patients with cleft lip and palate are currently undergoing treatment with our automated plate designs. These results indicate that our method could be suitable for global clinical use, which could facilitate access to this presurgical treatment, especially in low-income countries.

As future work, our registration step could be extended with deep learning to speed it up during inference. Such deep learning models can additionally serve as a stronger prior during registration by recognizing more distant correspondences when large deformations are required [20]. Finally, the customizability of our pipeline could be increased and its application generalized, which might include an extension to more cleft types, such as isolated cleft palate and Pierre Robin sequence, and to NAM devices. These and other options to be added to our pipeline will require feedback from a wider medical community.

## Supplementary Information

Below is the link to the electronic supplementary material.

Supplementary file 1 (pdf 5177 KB)

Supplementary file 2 (m4v 487002 KB)

## Footnotes

1 Intraoral Scanner i500 (Medit Corp., Korea).

2 Form 3B 3D printer using BioMed Clear Resin (Formlabs, USA).

## Declarations

### Content for publication

The parents have given their consent for the use of the image material presented in Fig. 5.
