# Supplementary material for: Automated and data-driven plate computation for presurgical cleft lip and palate treatment
Source: Int J Comput Assist Radiol Surg. 2023 Apr 2;18(6):1119–25. doi: 10.1007/s11548-023-02858-6 (PMC10284965; doi:10.1007/s11548-023-02858-6)
Supplement: Supplementary file 1 — (pdf 5177 KB) [file 11548_2023_2858_MOESM1_ESM.pdf]

Automated and Data-Driven Plate Computation for Presurgical Cleft Lip and Palate Treatment – Supplementary

Till N. Schnabel[1*], Baran Gözcü[1], Paulo Gotardo[2], Lasse Lingens[1], Daniel Dorda[1], Frawa Vetterli[1], Ashraf Emhemmed[1], Prasad Nalabothu[3,4,5], Yoriko Lill[3,4,5], Benito K. Benitez[3,4,5], Andreas A. Mueller[3,4,5], Markus Gross[1,2] and Barbara Solenthaler[1]

[1*]Department of Computer Science, ETH Zurich, 8092, Zurich, Switzerland.
[2]DisneyResearch|Studios, 8006, Zurich, Switzerland.
[3]Oral and Craniomaxillofacial Surgery, University Hospital Basel and University of Basel, 4031, Basel, Switzerland.
[4]Department of Clinical Research, University of Basel, 4031, Basel, Switzerland.
[5]Department of Biomedical Engineering, University of Basel, 4123, Allschwil, Switzerland.

*Corresponding author. E-mail: till.schnabel@inf.ethz.ch;

1 Automated Mesh Landmarking

This section provides more details about our deep-learning-based 3D landmarking method which forms the first component of our pipeline employed to align the raw input scan with the template and to initially guide the nonrigid surface registration. After discussing the related work for 3D mesh landmarking methods (subsection 1.1), more training details and quantitative results are provided in subsection 1.2 and 1.3 respectively.

1.1 Related Work

While classical landmarking approaches have shown promising results on constrained dental models by using fixed conditions, such as finding the peak points [1], and 2D-image-based landmark training has been thoroughly explored [2], robust automated landmarking on raw 3D scans has been lagging behind, as voxels require excessive memory for high resolution inputs and default neural network architectures are not suited for topology or representation changes [3]. PointNet and its variations [4, 5] have pioneered learning on unstructured 3D data and have already been successfully applied to landmarking applications on palate impressions [6], but their accuracy can be generally lower than that of models considering the surface connectivity defined by meshes, and the data amount required for training may be higher. DiffusionNet [7] is the first model that supports efficient deep learning on 3D surfaces of arbitrary representation, which makes it superior to other graph-based approaches like MeshCNN [8]. Since Sharp *et al.* [7] do not discuss the problem of landmarking, their work is extended in [9] by defining landmarking as a segmentation problem. The authors train a global DiffusionNet model in combination with an ensemble of localized models on predicting a full vertex segmentation map for each landmark with only vertices close to the landmark marked as non-zero. However, they do not provide a competitive performance for inputs of arbitrary orientation and, thus, focus their results on pre-aligned meshes. Since the computation of

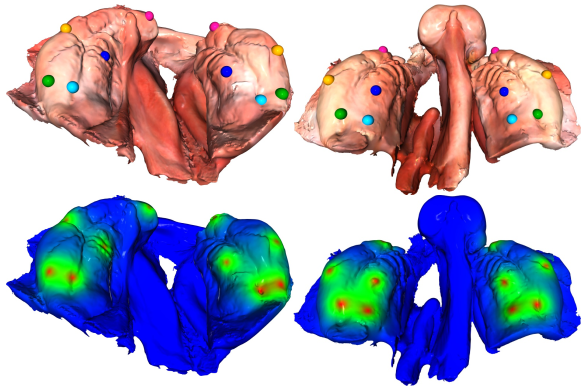

Fig. 1 We illustrate the reference landmark positions for a UCLP (left) and BCLP (right) intraorally-scanned mesh in the top row. For each landmark, DiffusionNet is fed a separate sparsely activated segmentation channel with an exponential fall-off around each landmark. The combination of all segmentation channels is illustrated in the bottom row.

such a pre-alignment generally requires some prior information about the input, we focus our results on inputs or arbitrary orientation. We employ an ensemble of two globally trained models, of which the first serves for aligning the input meshes to enable the second model to predict landmarks on an aligned mesh resulting in a similar precision as in [9]. Hence, our two-stage model is capable of processing raw input scans without requiring the user to provide any prior information.

1.2 Training Details

As described in the main document, our dataset comprises 283 UCLP and 114 BCLP scans, of which each was manually annotated with a set of 10 landmarks $\mathcal{L}_g$. Figure 1 illustrates the positions of these landmarks for a UCLP and BCLP scan with both alveolar segments being landmarked in the same following fashion:

1. Most anterior point on top of the crest (pink).
2. Lateral sulcus vertex/canine point on top of the crest (orange).
3. Crest point marking the appliance's posterior end (green).
4. Sulcus point marking the appliance's posterior end (light blue).
5. Cleft region/lift-off start on the same horizontal plane as landmark 2 (dark blue).

For BCLP meshes, landmark 3 is moved to position 4, whereas landmark 4 is moved inwards horizontally to mark the cleft region/lift-off start. This landmarking scheme was determined empirically to optimally guide the nonrigid registration. Representing the landmark positions $\mathcal{L}_g$ as continuous, sparsely activated segmentation maps $s_{i,j}$ (cf. main document), we train all DiffusionNet models with random mesh flipping to support input meshes of varying cleft sides. Additionally, the first-stage model is trained with fully randomized rotations to cover the whole spherical space of possible input orientations. The second-stage model, on the other hand, is trained on the pre-aligned input meshes either without any rotation randomization or on a limited azimuthal range of $\pm 0.2\,\text{rad}$ around the z-axis. The model uses 3D vertex coordinates (XYZ) as input features instead of the rotation-invariant 16-component heat kernel signature (HKS) vector proposed by Sharp *et al.* [7], since we observed inferior performance of HKS compared to XYZ, even with randomized rotations. As the majority of our dataset comprises uniformly colored plaster cast scans, we did not observe any benefit from concatenating vertex color information to the input feature vector, as proposed by [9]. However, a larger set of colored intraoral scans might yield different results. As already outlined in the main document, our models use the default parameters proposed by Sharp *et al.* [7] for segmentation purposes, except for an added dropout percentage of 0.2, since no significant benefit was observed when fine-tuning the hyperparameters. Training with the mean squared error (MSE) loss using an 80/20 training-test split converged after approximately 500 epochs, which took less than a day on an NVIDIA RTX 2080 TI. The number of mesh triangles required capping at 800 k to stay within the GPU's 11 GB memory limit.

1.3 Quantitative Evaluation

Our first landmark prediction tests concern the model's rotation variance. For this purpose, Table 1 compares the test accuracy of a model trained on pre-aligned meshes only (Fixed), i.e., without any rotation randomization, to one trained on completely random orientations (Random). The mean comparison suggests that a DiffusionNet model trained on fixed orientations is more accurate in its predictions, which is in line

Table 1 Automated landmark prediction accuracy [mm] on the test set, compared between the Fixed model trained on a fixed mesh orientation, the rotation-invariant HKS model, and the Random model trained on random orientations. For each mesh, the Random model's accuracy was measured for 10 random rotations, yielding the mean and standard deviation between the different orientations. The mean and standard deviation values were then averaged over all UCLP and BCLP meshes in the test set, yielding a separate mean and standard deviation for each value. Since the Fixed and HKS models are not tested on different orientations, they have zero StDev values.

	UCLP		BCLP	
	Mean	StDev	Mean	StDev
Fixed	1.58 ± 1.83	0	1.70 ± 1.34	0
HKS	5.32 ± 8.24	0	5.78 ± 8.53	0
Random	2.01 ± 1.27	1.23 ± 1.05	2.11 ± 1.35	1.12 ± 1.22

Table 2 Comparison of the prediction accuracy [mm] between three different model settings applied to 10 random rotations of each test mesh. The first model corresponds to the Random model from Table 1. The change in standard deviation is due to the value being averaged over both, the 10 random rotations and all test meshes.

	UCLP	BCLP
Single	2.01 ± 2.06	2.11 ± 2.13
Two-Stage-Fixed	1.81 ± 1.98	1.88 ± 1.55
Two-Stage-Varied	1.69 ± 1.85	1.70 ± 1.28

with the findings from [9]. Moreover, the non-zero prediction standard deviation of the Random model, which represents the prediction variation over different mesh orientations, shows that the model has not learned to be rotation-invariant despite having observed approximately 500 different orientations of each mesh during training. Possibly, the low number of training meshes (283 UCLP and 114 BCLP) is not enough to cover the large space of 3D random rotations sufficiently densely. Furthermore, we did not manage to achieve a competitive setting using the HKS input features, which Sharp *et al.* [7] proposed to overcome this issue of rotation variance. Table 1 shows our best setting with HKS input features. This setting employs a fixed input value thresholding, since we observed that the model predictions were prone to be disturbed by boundaries and spikes in the input mesh, possibly due to the high HKS feature values in these regions. Further investigations would be required to determine the optimal input representation for training on this specific dataset.

Table 2 lists a short ablation study to test our two-stage model setting, which aims at improving the prediction accuracy for random input orientations. The results were again averaged over 10 random rotations to eliminate any orientation bias. The first setting only applies the single Random model from Table 1 trained on fully randomized rotations. The other two rows report the results when utilizing this first model's landmark prediction $\mathcal{L}_{p_1}$ for a rough alignment via weighted Procrustes alignment [10], and then in a second step applying either the model trained on fixed orientations (Two-Stage-Fixed; this second model is the Fixed model from Table 1), or the other model trained on meshes with only slight rotation variations (Two-Stage-Varied). The numbers suggest that the second-stage model improves the prediction accuracy of the first-stage model on average. Moreover, the second-stage model seems to benefit from still observing small orientation variations during training. We believe that this decreases the model's overfitting capability and additionally compensates for the possibly inaccurate alignment given by the coarser landmark prediction of the first-stage model. These results from the Two-Stage-Varied model are comparable to those given by the Fixed model from Table 1 trained on and applied to meshes with fixed orientation. This indicates that the performance decrease caused by random input orientations can be compensated to some extent by our proposed two-stage model prediction, although complete rotation invariance is not achieved.

Finally, we test the intra- and inter-observer landmarking variance on the same 12 scans we printed for the model-based study in the main document. The scans were landmarked two times on separate days by the engineer who had already landmarked the whole dataset for an optimized

Table 3 Intra- and inter-observer euclidean landmark distance [mm].

	Intra-Observer	Inter-Observer	
	Engineer	Surgeon	Automated
UCLP	1.09 ± 0.84	1.95 ± 0.90	1.53 ± 0.93
BCLP	1.29 ± 0.69	1.87 ± 1.03	1.30 ± 0.62

registration performance without possessing any clinical knowledge. Additionally, a cleft surgeon provided a second set of landmarks, utilizing clinical knowledge to identify the necessary anatomical key points. These manual landmark positions are compared to the DiffusionNet predictions (Two-Stage-Varied from Table 2) in Table 3. The accuracy of the predicted landmarks approaches the intra-observer variance and is consistently better than that of the cleft surgeon, which indicates that the model has learned the engineer's specific landmarking scheme that may not strictly follow the anatomical description provided in the previous subsection.

2 Mesh Offsetting

We discuss the problem of mesh offsetting that we face during surface volumization and describe our convex smoothing solution to avoid triangle self-intersections.

2.1 Background

First, we briefly explain the concept of Laplacian smoothing as a prerequisite to our convex smoothing algorithm. Then, we describe the general problem of mesh offsetting and previous related work.

2.1.1 Laplacian Smoothing

Laplacian smoothing [11, 12] filters out noise and high-frequency details from a mesh while preserving the low-frequency structure, such as the general shape, as much as possible. In an iterative process, each vertex is adjusted towards the average of its neighbors by applying the forward Euler step

$$\mathbf{v}_i^{(n+1)} = \mathbf{v}_i^{(n)} + \lambda \left(\frac{1}{|\mathcal{N}_i|} \sum_{j \in \mathcal{N}_i} \mathbf{v}_j^{(n)} - \mathbf{v}_i^{(n)} \right), \quad (1)$$

where $\mathbf{v}_i^{(n)}$ refers to the vertex with index i at iteration n, $\mathcal{N}_i$ denotes the set of neighbors of vertex $\mathbf{v}_i^{(n)}$, and λ is a scale factor typically chosen in the range $(0, 1)$. Applying more and more iterations can lead not only to a loss of high frequency, but also to major mesh shrinkage if no additional constraints are applied. Selective Laplacian smoothing is a common technique where only a subset of the vertices $\mathcal{S} \subset \mathcal{V}$ is adjusted freely as in (1) while the boundary vertices that surround $\mathcal{S}$ serve as a constraint to keep the mesh from shrinking [13]. Likewise, we constrain the Laplacian adjustment vector per vertex to meet our conditions.

2.1.2 Problem

As illustrated in Figure 2, when offsetting a 1D function, the offset distance must not exceed the curvature radius $r = \frac{1}{k}$ at any point, i.e., the inverse of the curvature k, to avoid that the offset function overlaps around this region. On a surface function, such overlapping may happen in multiple directions. Therefore, the minimum principal curvature k_1 defines the limit of the offsetting distance. Mesh offsetting describes the problem of offsetting a general surface, discretized as a mesh, by an arbitrarily desired distance without causing any overlaps, i.e., self-intersections, in convex regions.

2.1.3 Related Work

Early work treated this mesh offsetting problem as a Minkowski sum of faces, cylinders for edges, and spheres for vertices. Whereas point-based resampling [14] showed only limited application without properly discussing self-intersections, curve-based resampling [15] relied on analytic curve estimation, which requires 2D slicing and is computationally inefficient. Other literature attempted to split vertices on sharp edges using the multiple normal directions defined by their surrounding faces [16], which requires a closed 2D manifold surface. More recent works represent the surface implicitly via signed distance functions [17], which allows resampling the surface at an arbitrary offset distance without any self-intersections, potentially with the use of marching cubes [18]. However, none of these works preserve the mesh topology of the surface to be offset, which may complicate further automated mesh processing. Hence,

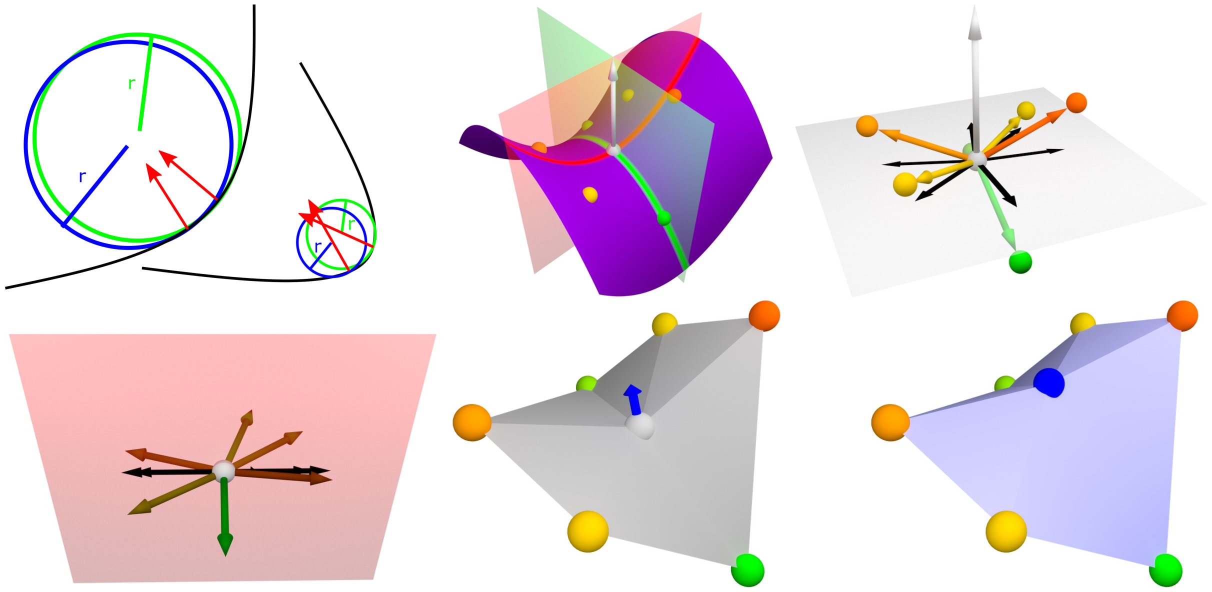

Fig. 2 In the top row, the left image illustrates the offsetting problem in 2D. The higher the curvature $k = \frac{1}{r}$ is, the less the function can be offset without any overlaps. The middle image shows the orthogonal principal curvatures $\mathbf{k_1}$ and $\mathbf{k_2}$ (red and green plane) defining the problem in 3D. The colored spheres represent the discretized neighbors of the central vertex in white. A standard Laplacian update step would not adjust the central vertex in positive normal direction to reduce $\mathbf{k_1}$, since the green vertices would pull it in the opposite direction. The right image demonstrates how our convex smoothing algorithm improves this adjustment by projecting the neighbor connection vectors onto the white normal plane and then onto the $\mathbf{k_1}$ plane in the bottom row left image. Finally, the last two images visualize how the vertex is adjusted in positive normal direction as an average of the neighbors weighted by the magnitude of their corresponding projected connection vectors.

we developed a novel offsetting algorithm that reduces the curvature selectively via customized Laplacian smoothing, enabling preservation of the original mesh topology.

2.2 Convex Smoothing

Our convex smoothing algorithm globally raises k_1 above the required threshold k_t to enable offsetting a surface by a desired distance. We adopt libigl's [18] principal curvature approximation per vertex [19] for the discretization of a surface via a triangle mesh $\mathcal{M}$ that consists of a set of vertices $\mathcal{V}$ and triangles $\mathcal{T}$. The vertices with $k_1 < k_t$ are adjusted via weighted Laplacian smoothing, where the vertex neighbors are weighted according to their parallelity with the direction $\mathbf{k_1}$ of the minimum principal curvature. More precisely, the connection vector $\mathbf{v}_{ij}$ between the vertex $\mathbf{v}_i$ and each neighbor $\mathbf{v}_j \in \mathcal{N}_i$ is projected onto the plane $A_{\mathbf{v}_i}$ perpendicular to $\mathbf{v}_i$'s normal. Afterwards, the weight $w_{\mathbf{v}_j}$ of neighbor $\mathbf{v}_j$ corresponds to the absolute dot product between this projected and normalized connection vector $\hat{\mathbf{v}}_{j,p}$ and the minimum principal curvature direction $\mathbf{k_1}$. Lastly, the weights of all neighbors $w_{\mathbf{v}_j}$ are rescaled to sum to 1. Figure 2 summarizes this convex smoothing procedure, and Algorithm 1 specifies the corresponding algorithm. The set of boundary vertices $\mathcal{B}$ is excluded, as the curvature is not well-defined on the boundary and a proper adjustment of the remaining vertices is sufficient to eliminate any self-intersections. Note that, when offsetting the vertices in the opposite direction, the algorithm works analogously by reducing the maximum curvature instead of increasing the minimum curvature to ensure that $k_2 < k_t$. Since this algorithm does not require resampling, it preserves the mesh surface topology and thus also the corresponding segmentation, which facilitates further automated processing during the plate computation.

Algorithm 1 Convex Smoothing Algorithm

Input: Triangle mesh $\mathcal{M} = (\mathcal{V}, \mathcal{T})$
Output: Convexly smoothed mesh $\mathcal{M}' = (\mathcal{V}', \mathcal{T})$

for iterations **do**
  for all $\mathbf{v}_i \in \mathcal{V} \setminus \mathcal{B}$ **do**
    if $k_1(\mathbf{v}_i) < k_t$ **then**
      for all $\mathbf{v}_j \in \mathcal{N}_i$ **do**
        $\mathbf{v}_{j,p} \leftarrow$ project $(\mathbf{v}_j - \mathbf{v}_i)$ to $A_{\mathbf{v}_i}$
        $w_{\mathbf{v}_j} \leftarrow \left|\frac{\mathbf{v}_{j,p} - \mathbf{v}_i}{\|\mathbf{v}_{j,p} - \mathbf{v}_i\|_2} \cdot \frac{\mathbf{k_1}}{\|\mathbf{k_1}\|_2}\right|$
      end for
      for all $\mathbf{v}_j \in \mathcal{N}_i$ **do**
        $\mathbf{v}_i \leftarrow \mathbf{v}_i + \frac{w_{\mathbf{v}_j}}{\sum_j w_{\mathbf{v}_j}}(\mathbf{v}_j - \mathbf{v}_i)$
      end for
    end if
  end for
end for

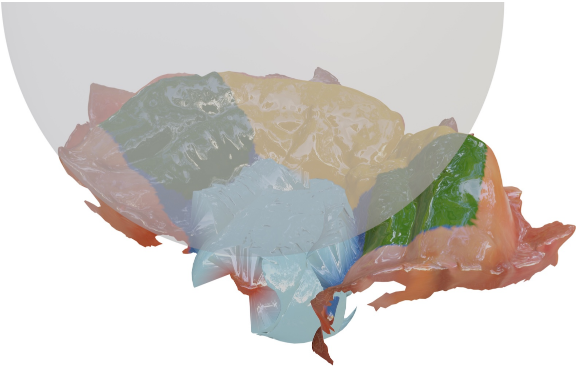

Fig. 3 We show our sphere prediction (gray) as the solution to our optimization problem defined in (2). The colored input scan, shown from the posterior, has the left and right ridge $\mathcal{S}_l, \mathcal{S}_r$ marked in green, the area $\mathcal{S}_o$ we minimize our objective function on marked in blue and green, and the constraining area $\mathcal{S}_c$ marked by all added colors. The points marked in light blue are offset by 2.5 mm in order to maintain the required safety distance to the inner cleft palate tissue.

3 Palate Sphere Approximation

As discussed in the main document, the cleft palate area should be covered by a plate that imitates the round shape of a healthy palate [20]. In this section, we describe the sphere we predict on top of the cleft palate mesh data to approximate such a healthy palate. The four parameters of the sphere are derived via a Voronoi diagram, which can be used to find the locally largest empty spheres [21]. The vertices of the input mesh in the inner cleft palate area are offset by 2.5 mm to ensure that the required safety distance is kept everywhere. Afterwards, the Voronoi diagram is constructed on a larger set of constraining points, which yields a finite set of spheres of which none penetrates the input mesh. We only consider those spheres that touch both the left and the right alveolar ridge and choose the optimal sphere that minimizes the overall distance to all points. Formally, we thus minimize the constrained least-squares objective

$$\begin{aligned} \min_{\mathbf{c},r} \sum_{i\in\mathcal{S}_o} (\|\mathbf{c}-\mathbf{v}_i\|_2 - r)^2, & \\ \text{s.t. } \|\mathbf{c}-\mathbf{v}_i\|_2 \geq r \quad & \forall i \in \mathcal{S}_c, \\ \|\mathbf{c}-\mathbf{v}_j\|_2 = r \quad & \exists j \in \mathcal{S}_l, \\ \|\mathbf{c}-\mathbf{v}_k\|_2 = r \quad & \exists k \in \mathcal{S}_r, \end{aligned} \tag{2}$$

where the sphere parameters $\mathbf{c}, r$ denote the center and radius respectively, $\mathbf{v}$ denotes a vertex of the input scan, $\mathcal{S}_c$ denotes the constraining set of all points the sphere must not penetrate, $\mathcal{S}_o \subset \mathcal{S}_c$ the set of vertices in the cleft palate region and both alveolar ridges, and $\mathcal{S}_l, \mathcal{S}_r \subset \mathcal{S}_o$ the points on the left and right alveolar ridge respectively. The approximation via largest empty spheres is crucial, as this problem is generally non-convex. Figure 3 visualizes the concept of this sphere prediction.

4 Implementation Details

We provide details about the implementation of our pipeline and the corresponding GUI. The pipeline was implemented in Python 3.9 [22]. Among other packages, the code relies on Open3D [23] and trimesh [24] for geometry processing, PyTorch [25] for the landmark prediction, libigl [18] for the computation of the principal curvatures, and Menpo [26] for an efficient implementation of nonrigid iterative closest point (NICP) [27]. We adjusted the NICP implementation to ensure efficient Windows compatibility. Additionally, the whole pipeline only performs CPU computations to offer maximum flexibility towards different operating systems and end devices. The GUI, illustrated in Figure 4, is implemented in Blender 3.0 [28] and has been tested on several devices running Windows, Ubuntu, and MacOS (Intel and Apple Silicon). All packages are automatically installed, allowing for minimal user installation effort. The workflow with the GUI comprises importing a palatal scan, predicting the landmarks for this

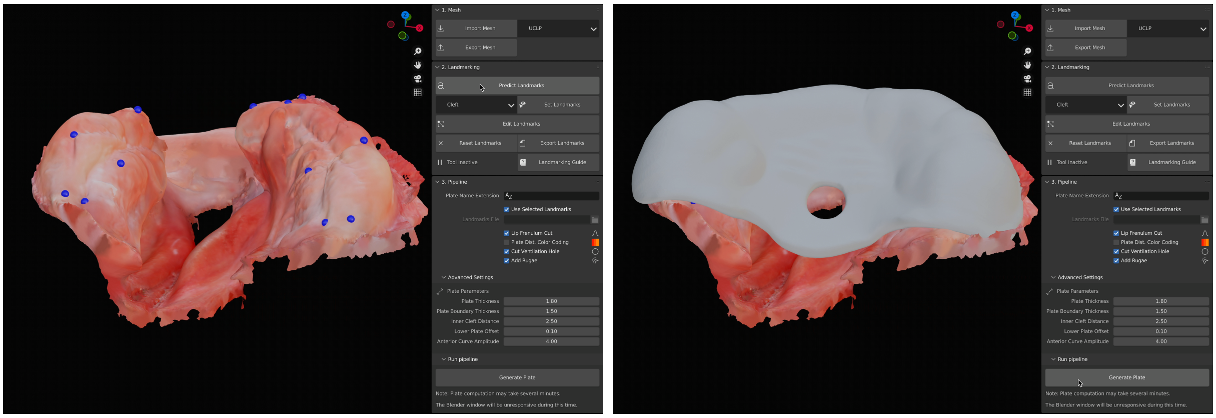

Fig. 4 Captures of our Blender-based GUI. After automatically predicting the landmarks on a UCLP or BCLP mesh, the plate can be computed, with optional adjustments to the shape including addition of stimulation elements and a ventilation hole.

scan, optionally adjusting the default parameters if a different plate thickness is desired or a ventilation or stimulation elements should be added, and finally computing the plate. Online Resource 2 demonstrates this workflow in a video, where the pure computations, given a high resolution intraoral scan as input, take less than 2 min on a 14-inch Apple MacBook Pro with an 8-core M1 chip and 16 GB RAM. The computation time for the landmark prediction and the subsequent surface registration may vary depending on the input size, and machines with a less powerful CPU may take approximately 3 min for the same computations. However, GPU utilization and optimization could be implemented to increase the performance further.

References

[1] Woodsend, B., Koufoudaki, E., Mossey, P.A., Lin, P.: Automatic recognition of landmarks on digital dental models. Comput Biol Med **137**, 104819 (2021). https://doi.org/10.1016/j.compbiomed.2021.104819

[2] Johnston, B., Chazal, P.d.: A review of image-based automatic facial landmark identification techniques. EURASIP J Image Video Processing **2018**(1), 86 (2018). https://doi.org/10.1186/s13640-018-0324-4

[3] Maturana, D., Scherer, S.: Voxnet: A 3d convolutional neural network for real-time object recognition. In: 2015 IEEE/RSJ International Conference on Intelligent Robots and Systems (IROS), pp. 922–928 (2015). https://doi.org/10.1109/IROS.2015.7353481

[4] Qi, C.R., Su, H., Mo, K., Guibas, L.J.: Pointnet: Deep learning on point sets for 3d classification and segmentation. In: 2017 IEEE Conference on Computer Vision and Pattern Recognition (CVPR), pp. 77–85 (2017). https://doi.org/10.1109/CVPR.2017.16

[5] Qi, C.R., Yi, L., Su, H., Guibas, L.J.: Pointnet++: Deep hierarchical feature learning on point sets in a metric space. NIPS'17, pp. 5105–5114. Curran Associates Inc., Red Hook, NY, USA (2017). https://doi.org/10.48550/arXiv.1706.02413

[6] Croquet, B., Matthews, H., Mertens, J., Fan, Y., Nauwelaers, N., Mahdi, S., Hoskens, H., El Sergani, A., Xu, T., Vandermeulen, D., Bronstein, M., Marazita, M., Weinberg, S., Claes, P.: Automated landmarking for palatal shape analysis using geometric deep learning. Orthod Craniofac Res **24 Suppl 2**, 144–152 (2021). https://doi.org/10.1111/ocr.12513

[7] Sharp, N., Attaiki, S., Crane, K., Ovsjanikov, M.: Diffusionnet: Discretization agnostic learning on surfaces. ACM Transactions on Graphics **41**, 1–16 (2022). https://doi.org/10.1145/3507905

[8] Hanocka, R., Hertz, A., Fish, N., Giryes, R.,

Fleishman, S., Cohen-Or, D.: Meshcnn: A network with an edge **38**(4) (2019). https://doi.org/10.1145/3306346.3322959

[9] Carotenuto, L.: Three dimensional facial landmark detection in 3d photos. Master's thesis, Radboud University (2022). Accessed: 2022-08-19. https://www.ai-for-health.nl/projects/3d-landmark-detection

[10] Gower, J.C.: Generalized procrustes analysis. Psychometrika **40**(1), 33–51 (1975). https://doi.org/10.1007/BF02291478

[11] Herrmann, L.R.: Laplacian-isoparametric grid generation scheme. J Eng Mech **102**(5), 749–756 (1976). https://doi.org/10.1061/JMCEA3.0002158

[12] Sorkine, O., Cohen-Or, D., Lipman, Y., Alexa, M., Rössl, C., Seidel, H.-P.: Laplacian surface editing. In: Proceedings of the 2004 Eurographics/ACM SIGGRAPH Symposium on Geometry Processing. SGP '04, pp. 175–184. Association for Computing Machinery, New York, NY, USA (2004). https://doi.org/10.1145/1057432.1057456

[13] Tutte, W.T.: How to draw a graph. Proc London Math Soc **s3-13**(1), 743–767 (1963). https://doi.org/10.1112/plms/s3-13.1.743

[14] Chen, Y., Wang, H., Rosen, D.W., Rossignac, J.: A point-based offsetting method of polygonal meshes. ASME Journal of Computing and Information Science in Engineering **1**, 21 (2005)

[15] Jun, C.-S., Kim, D.-S., Park, S.: A new curve-based approach to polyhedral machining. Computer-Aided Design **34**(5), 379–389 (2002). https://doi.org/10.1016/S0010-4485(01)00110-5

[16] Kim, S.-J., Lee, D.-Y., Yang, M.-Y.: Offset triangular mesh using the multiple normal vectors of a vertex. Computer-Aided Design and Applications **1**(1-4), 285–291 (2004). https://doi.org/10.1080/16864360.2004.10738269

[17] C.L. Wang, C., Chen, Y.: Thickening freeform surfaces for solid fabrication. Rapid Prototyping Journal **19**(6), 395–406 (2013). https://doi.org/10.1108/RPJ-02-2012-0013

[18] Jacobson, A., Panozzo, D., et al.: libigl: A simple C++ geometry processing library. https://libigl.github.io/. (accessed 30 June 2022) (2018)

[19] Panozzo, D., Puppo, E., Rocca, L.: Efficient multi-scale curvature and crease estimation. 2nd International Workshop on Computer Graphics, Computer Vision and Mathematics, GraVisMa 2010 - Workshop Proceedings, pp. 9–16 (2010)

[20] Hohoff, A., Stamm, T., Meyer, U., Wiechmann, D., Ehmer, U.: Objective growth monitoring of the maxilla in full term infants. Arch Oral Biol **51**(3), 222–235 (2006). https://doi.org/10.1016/j.archoralbio.2005.07.007

[21] Shamos, M.I.: Computational Geometry. Yale University, New Haven, CT, USA (1978). https://doi.org/10.1007/978-1-4612-1098-6

[22] Van Rossum, G., Drake, F.L.: Python 3 Reference Manual. CreateSpace, Scotts Valley, CA (2009)

[23] Zhou, Q.-Y., Park, J., Koltun, V.: Open3D: A modern library for 3D data processing. arXiv:1801.09847 (2018)

[24] Dawson-Haggerty et al.: Trimesh. https://trimsh.org/

[25] Paszke, A., Gross, S., Chintala, S., Chanan, G., Yang, E., DeVito, Z., Lin, Z., Desmaison, A., Antiga, L., Lerer, A.: Automatic differentiation in pytorch. In: NIPS-W (2017)

[26] Alabort-i-Medina, J., Antonakos, E., Booth, J., Snape, P., Zafeiriou, S.: Menpo: A comprehensive platform for parametric image alignment and visual deformable models. In: Proceedings of the 22nd ACM International Conference on Multimedia. MM '14, pp. 679–682. Association for Computing Machinery, New York, NY, USA (2014). https://doi.org/10.1145/2647868.2654890

[27] Amberg, B., Romdhani, S., Vetter, T.: Optimal step nonrigid icp algorithms for surface registration. In: IEEE Conf Comput Vis Pattern Recognit (CVPR), pp. 1–8 (2007). https://doi.org/10.1109/CVPR.2007.383165

[28] Blender Online Community: Blender - a 3D Modelling and Rendering Package. Blender Foundation, Stichting Blender Foundation, Amsterdam (2018). Blender Foundation. http://www.blender.org
